# Acute myocardial infarction in the elderly

**DOI:** 10.1007/s12471-015-0751-0

**Published:** 2015-09-17

**Authors:** F. Zijlstra, M.-J. de Boer

**Affiliations:** 1 Department of Cardiology, Thoraxcenter, Erasmus University Medical Center, Room Ba 593, ’s- Gravendijkwal 230, PO Box 2040, 3000 CA Rotterdam, The Netherlands; 2 Department of Cardiology Radboud University Medical Center Nijmegen, Nijmegen, The Netherlands

The elderly are the fastest growing part of the population in Western countries and aged individuals constitute a rapidly increasing proportion of patients presenting with acute coronary syndromes (ACS), including ST-elevation myocardial infarction (STEMI). Advanced age is a strong predictor of adverse outcome. Unfortunately, most reported ACS trials have included limited numbers of patients over 75 or 80 years of age, and therefore limited data are available on the best management of this growing subset of the ACS population. Recommended therapies are sometimes withheld in very old individuals, due to lack of proof of benefit for this age group and the high risk of serious side effects and complications. This is, at least in part, also due to specific clinical characteristics of these patients at initial presentation. Symptoms following acute coronary occlusion are less specific, electrocardiographic patterns more often not the typical pattern of ST-segment elevation and confounding morbidity may all contribute to diagnostic uncertainty and delayed or ‘conservative’ decision-making.

In this issue of the Netherlands Heart Journal, Claessen et al. attempt to fill in the gaps in our knowledge, by a detailed description of a cohort of 196 patients aged ≥ 80 years as part of the 2002 patients treated with primary angioplasty for STEMI at the Academic Medical Center Amsterdam between 1 January 2003 and 31 July 2008 [1]. At 3-year follow-up, mortality and the incidence of both ischaemic and bleeding events were strongly related to age. With a 3-year mortality of ± 40 %, reinfarction in ± 23 %, bleeding in 1/3 of patients and stroke in 1/12 of patients ≥ 80 years, it is clear that there is a large potential for improvement and it is certainly appropriate to give this problem a more prominent place on our scientific agenda. However, before embarking on the collection and description of more registry data, or even, as the authors suggest, a dedicated randomised controlled trial, we should consider some issues to place these findings in perspective.

## Patient selection

To be included in this cohort, the elderly STEMI patient had to reach the cathlab and they had to survive the primary angioplasty procedure. In a paper entitled ‘Changing trends in, and characteristics associated with, NOT undergoing cardiac catheterisation in elderly adults hospitalised with ST-segment elevation acute myocardial infarction’, Tisminetzky *et al.* describe the decade long (1999–2009) trends in the rate of NOT undergoing angiography and angioplasty and the factors associated with not undergoing these procedures in an observational population-based study in the setting of Worchester, Massachusetts [2]. Older adults who develop STEMI are increasingly likely to undergo cardiac catheterisation and angioplasty, but several high-risk groups often still do not make it to the cathlab, including women, individuals with prior infarction and those with various comorbidities. From a population perspective outcomes are likely to be considerably worse compared with the patients described by the AMC investigators.

## If patients make it to the cathlab, are we doing better?

In a report from the Swedish coronary angiography and angioplasty registry (SCAAR), Velders et al. describe 4876 elderly STEMI patients [3]. Procedural success and prognoses of these patients > 80 years remained similar during a 10-year period, despite changes in patient characteristics and treatment. The good news of this report is that although advanced age strongly increased the risk of adverse events, survivors of the early phase had a slightly improved prognosis compared with the general population. However, this can also be interpreted as further evidence for patient selection bias and undertreatment.

## Bleeding complications

Access site related as well as non-access related bleeding has a negative impact on quality of life and is related to short- and long-term prognosis. In particular, gastrointestinal bleeding is related to a number of risk factors including age and the use of triple antithrombotic therapy (oral anticoagulation + dual antiplatelet therapy) and is a strong independent predictor of all-cause mortality at 1 year [4].

## Implications

The authors conclude that ‘especially in this high-risk patient group individualised therapy is needed to optimise clinical outcomes’. We will attempt to be more specific.

- **Presentation:** We should be aware that acute coronary occlusion in elderly patients often results in ‘atypical’ clinical presentations, in particular in women, and that the sensitivity and specificity of the electrocardiographic changes in these patients are modest, resulting in a risk of underdiagnosis.
- **Decision-making:** Although ischaemic time is important in some STEMI patients, in the elderly the impact of a 30 or even 60 minute delay is very modest or even absent [5]. See also for instance Tables 3 and 4 of the Supplementary Appendix of the Claessen paper. All elderly patients with suspected ACS should therefore undergo a thorough clinical evaluation prior to a decision to proceed or not to proceed to the cathlab.
- **Pharmacotherapy:** As heparin is difficult to dose, in particular in the elderly, measure ACT or APTT, avoid overdosing and be aware that many patients receive heparin in the ambulance. If at all possible do not use glycoprotein IIb/IIIa inhibitors. In view of the relatively high incidence of atrial fibrillation in the elderly, do not discharge patients with oral anticoagulation + dual antiplatelet therapy, but with oral anticoagulation + clopidogrel [6]
- **In the cathlab:** go radial and keep it simple: stent the ruptured plaque, not the entire infarct-related artery. As long as you do not know the renal function, restrict contrast use. The incidence of acute heart failure and respiratory failure in these patients is considerable, be certain that somebody pays attention to the patient while you are focused on optimising the angiographic result of the primary angioplasty. As the majority of these patients have multivessel disease, consider staged procedures with fractional flow reserve guidance as the preferred option for revascularisation of non-infarct related arteries.

The AMC investigators deserve a compliment for the collection of detailed clinical data in combination with 3-year follow-up of a large contemporary cohort of elderly primary angioplasty patients. The topic certainly deserves more attention and on the basis of these data a collaborative effort (under auspices of ICIN?) to address the important remaining issues seems urgently needed.

### Funding

None.

### Conflict of interests

None declared.
